# Multi-Sensor Recursive EM Algorithm for Robust Identification of ARX Models

**DOI:** 10.3390/s25227060

**Published:** 2025-11-19

**Authors:** Xin Chen, Jiale Li

**Affiliations:** School of Electronics and Information Engineering, Suzhou University of Science and Technology, Suzhou 215009, China; leejiale.non@gmail.com

**Keywords:** robust system identification, multi-sensor data fusion, ARX model, recursive EM algorithm, student’s *t*-distribution

## Abstract

A robust multi-sensor recursive Expectation-Maximization (RMSREM) algorithm is proposed in this paper for autoregressive eXogenous (ARX) models, addressing the challenges of heavy-tailed noise, as well as the difficulty in simultaneously processing multi-sensor information. First, for the potential outliers in industrial processes, the Student’s *t*-distribution is introduced to model the statistical characteristics of measurement noise, whose heavy-tailed property enhances the algorithm’s robustness. Second, a recursive framework is integrated into the Expectation-Maximization (EM) algorithm to satisfy the real-time requirement of dynamic system identification. Through a recursive scheme of the *Q*-function and sufficient statistics, model parameters are updated in real-time, allowing them to adapt to time-varying system characteristics. Finally, by exploiting the redundancy and complementarity of multi-sensor data, a multi-sensor information fusion mechanism is designed that adaptively calculates the weight of each sensor based on the noise variances. This mechanism effectively fuses multi-source observation information and mitigates the impact of single-sensor failure or inaccuracy on identification performance. Numerical examples and simulations of the continuous stirred-tank reactor (CSTR) demonstrate the validity of the proposed RMSREM algorithm.

## 1. Introduction

System identification is a cornerstone of modern control theory and engineering applications. It aims to create mathematical models that can accurately describe the dynamic characteristics of a system using input-output data [1,2]. Among various identification model structures, the autoregressive with exogenous input (ARX) model has been widely applied in the fields of industrial processes, chemical production, and biological manufacturing, due to its concise structure and efficient computation [3,4]. In practical environments, the measurement signals are often affected by accidental factors, such as intense noise, gross errors, missing data, and sensor failure. The above abnormal disturbances are induced by latent variables that are not directly observable. The Expectation-Maximization (EM) algorithm [5] creates a practical statistical framework for solving maximum likelihood estimation problems involving latent variables or incomplete data. Meanwhile, multiple sets of sensors can be deployed to collect large amounts of measurement data, to improve identification accuracy [6]. Most existing methods for the identification of multiple sensors rely on the assumption of identically distributed data, which is not suitable for simultaneous multi-sensor processing. An urgent need for new identification methods appears nowadays [7,8].

Titterington et al. [9] pioneered the recursive EM algorithm (REM) via stochastic approximation, recursively updating parameters using observed data likelihood gradient and Fisher information matrix (FIM) of complete data. To avoid FIM inversion, Cappé et al. [10] investigated an online EM algorithm based on recursive sufficient statistics with a focus on exponential family distributions. Later, the online EM algorithm was applied to the parameter estimation of hidden Markov models in [11]. A recursive EM identification relying on sufficient statistics was developed in [12], where additional iterations were conducted for each time instant. Considering the time-delay, Guo et al. [13] employed REM and convex optimization for the identification of Markov jump autoregressive systems. A recursive parameter estimation approach for the Dirichlet hidden Markov models was developed in [14]. A Student’s *t*-distribution-based REM was developed in [15] for the robust identification of linear ARX models. The varying delay issue of an integrated measurement systems was considered in [16], where an online EM algorithm was employed for parameter learning. The robust recursive identification was developed for time-delay systems in [17] integrated with skewed measurement noise.

On the other hand, Multi-Sensor identification mainly involves two processing methods: data fusion and multi-task learning (MTL) [18,19]. Data fusion technology further improves data availability and redundancy through the extensive deployment of multi-sensor systems. Simultaneously observing the same process variable with multiple sensors can reduce the impact of single-sensor failure or anomalies, improving identification accuracy and reliability [20]. Relevant research is mainly divided into two streams: probabilistic statistical methods and artificial intelligence approaches [21]. Due to its simplicity and efficiency, the weighted fusion algorithm has become an ideal choice for fusing data of varying accuracies. The optimal and unbiased fused data can be obtained by reasonably evaluating weight coefficients in the weighted fusion algorithms [22]. Data fusion methods obtain high quality measurement data through weighted averaging or optimal fusion approaches [23], whereas MTL methods share information among sensors from the perspective of optimization objectives, improving overall performance [24,25]. However, some deficiencies still exist in current approaches. Although these methods assume different noise characteristics across multiple sensors, they mostly rely on the Gaussian noise assumption. As is well known, the Gaussian assumptions are sensitive to non-Gaussian perturbations and outliers. Once sensor measurements exhibit heavy-tailed distributions or sudden spike disturbances, their identification performance would rapidly degrade.

In industrial engineering practice, outliers often appear due to unknown reasons, such as sensor faults and signal transmission disturbances. Improper disposition of the outliers may lead to degradation in model performance. In engineering, the most general outlier processing methods are data trimming and smoothing. Although the data trimming and smoothing approaches are intuitive and easy to understand, the identification models will suffer from information loss and estimation bias [26]. To enhance the robustness of system identification in non-Gaussian noise environments, statistical modeling methods based on the *t*-distribution have attracted researchers’ attention. Compared to ordinary Gaussian distribution, *t*-distribution has heavier tails in its probability density function (pdf), which increases tolerance for outliers [27]. Due to remarkable reliability and analytical properties, the *t*-distribution has been widely adopted in identification fields. For example, a robust Bayesian technique for logistic regression modeling was proposed in [28], where a weakly informative Student’s t prior distribution was employed. Yu et al. [29] considered the joint estimation of states and noise covariance for linear systems with unknown covariance of multiplicative noise, where the measurements were modeled as a mixture of a Student’s *t*-distribution and Gaussian distributions. Several alternatives to the EM algorithm were explored in [30] for ML estimation of location, scatter matrix, and degree of freedom (dof) of the Student *t*-distribution. Beyond the maximum likelihood ideology, the application of the *t*-distribution has also been documented in numerous studies [27,31] within the ideology of variational Bayesian inference for identification tasks.

However, a robust multi-sensor fusion technology is still not receiving sufficient attention for online identification. Based on the above background, this paper introduces the concept of multi-sensor fusion in the online EM algorithm and proposes a robust multi-sensor recursive EM algorithm (RMSREM). On the one hand, the proposed algorithm can effectively fuse information from multi-source observations. On the other hand, RMSREM algorithm can enhance robustness against outliers via incorporating the *t*-distribution in noise modeling. Moreover, the recursive EM framework enables real-time updates of model parameters when new data arrives. With the implementation of the proposed method, the heterogeneous information from multiple sensors is adequately utilized. The identification accuracy is significantly improved. Moreover, the performance of the estimated models remains stable in complex environments, involving sensor plug-and-play, noise interference, and outliers. The main contributions of this article are given as follows:

- The Student’s *t*-distribution is incorporated in the algorithm to describe the statistical characteristics of measurement noise, whose heavy-tailed property promotes the algorithm’s robustness;
- Second, a recursive *Q*-function is derived, based on which a recursive framework of the EM algorithm is accomplished together with sufficient statistics recursion. The real-time requirement of dynamic system identification is satisfied;
- A multi-sensor information fusion mechanism is designed. Multi-source information is fused via adaptive calculation of the weight of each sensor. The reliability of the identification algorithm has been enhanced.

The structure of this paper is as follows: the preliminary concepts and background knowledge of the proposed RMSREM are introduced in Section 2, Section 3 provides the mathematical explanation of the robust multi-sensor recursive EM algorithm, Section 4 verifies the adaptability and efficacy of the proposed algorithm through a numerical example and simulations of the CSTR, and Section 5 presents the final research conclusions.

## 2. Problem Formulation

Considering a linear system with *M* sensors, one of the ARX model is defined as

(1) $$y_{k}^{m}={\left(x_{k}^{m}\right)}^{T}\theta +e_{k}^{m},\;k=1,2,\ldots ,N,$$

where *k* is the time index and m=1,2,…,M indicates the sensor number. The noisy observation obtained from the *m*-th sensor at instant *k* is denoted by $y_{k}^{m}$. The corresponding regressive vector is $x_{k}^{m}={\left[y_{k-1}^{m},\ldots ,y_{k-n_{a}}^{m},\;u_{k-1},\ldots ,u_{k-n_{b}}\right]}^{T}\in R^{n}$, which incorporates past outputs of the same sensor together with previous inputs. The system input at time *k* is represented by $u_{k}\in R^{1}$. The unknown parameter vector to be estimated is $\theta ={\left[a_{1},\ldots ,a_{n_{a}},\;b_{1},\ldots ,b_{n_{b}}\right]}^{T}\in R^{n}$, where $n_{a}$ and $n_{b}$ denote the output and input polynomial orders. The measurement noise from the *m*-th sensor is indicated by $e_{k}^{m}$, which is assumed to follow a Student’s *t*-distribution, i.e., $e_{k}^{m}∼t\left(\mu_{m},\sigma_{m}^{2},\nu_{m}\right).$

**Assumption** **1.** *The measurement noise $\left\{e_{k}^{m}\right\}$ from the same sensor are independent and identically distributed.*

**Assumption** **2.** *The measurement sets $\left\{y_{k}^{1},\ldots ,y_{k}^{M}\right\}$ are independent but not identically distributed.*

**Assumption** **3.** *For the sake of computational simplicity, the distinct distributions are defined as Student’s t-distributions with different variances and different dofs.*

The pdf of Student’s *t*-distribution is as follows:

(2) $$t\left(e_{k}^{m}|\mu_{m},\sigma_{m}^{2},\nu_{m}\right)=\frac{\Gamma \left(\frac{\nu_{m}+d}{2}\right){\left|\sigma_{m}^{2}\right|}^{-\frac{1}{2}}}{{\left(\pi \nu_{m}\right)}^{\frac{d}{2}}\Gamma \left(\frac{\nu_{m}}{2}\right){\left[1+\delta \left(e_{k}^{m}|\mu_{m},\sigma_{m}^{2}\right)/\nu_{m}\right]}^{\frac{\nu_{m}+d}{2}}},$$

where $\mu_{m}\in R^{1}$ represents the mean of the measurement noise (taken as $\mu_{m}=0$ in this work), $\sigma_{m}^{2}\in R^{1}$ is its variance, and $\nu_{m}\in R^{1}$ specifies the dof. The measurement dimension is denoted by $d\in N^{1}$, with d=1 in this case. Γ(t) is the gamma function. Furthermore, the square of Mahalanobis distance between the noise term $e_{k}^{m}$ and the mean value $\mu_{m}$, given the variance $\sigma_{m}^{2}$, is defined as $\delta \left(e_{k}^{m}|\mu_{m},\sigma_{m}^{2}\right)={\left(e_{k}^{m}-\mu_{m}\right)}^{2}/\sigma_{m}^{2}$.

In system identification and parameter estimation, it is conventional to assume that the noise follows a Gaussian distribution. The Gaussian assumption is primarily due to its analytical property and alignment with engineering practice. Nevertheless, Gaussian-based models are vulnerable to outliers and abnormal disturbances in complex environments. To alleviate such sensitivity, the so-called contaminated Gaussian model introduces a mixture of two Gaussian components with different variances, where the one with the larger variance is set to accommodate outliers. From a similar perspective, the Student’s *t*-distribution can be regarded as a limit case of Gaussian mixtures that share an identical mean but whose variances vary continuously from 0 to *∞*, governed by a variance-scaling mechanism [32]. By incorporating a latent weighting variable $r_{m,k}\in R^{1}$, which adjusts the influence of irregular deviations in the measurements, the probability of the measurement noise $e_{k}^{m}$ under the *t*-distribution can be reformulated as

(3) $$p\left(e_{k}^{m}|0,\sigma_{m}^{2},\nu_{m}\right)=\int p\left(e_{k}^{m}|0,\sigma_{m}^{2},r_{m,k}\right)p\left(r_{m,k}|\nu_{m}\right)dr_{m,k}.$$

where the process noise $e_{k}^{m}$, when re-scaled by the latent weight $r_{m,k}$, is subject to a conditional Gaussian distribution, namely $e_{k}^{m}|0,\sigma_{m}^{2},r_{m,k}∼N\;\left(0,\sigma_{m}^{2}/r_{m,k}\right).$ In parallel, the scaling variable $r_{m,k}$ is assumed to follow a Gamma distribution parameterized by the degrees of freedom $\nu_{m}$, that is $r_{m,k}|\nu_{m}∼G\;\left(\nu_{m}/2,\nu_{m}/2\right).$ An important property is that as $\nu_{m}\to \infty$, the distribution of $r_{m,k}$ collapses to the constant value 1, implying that the *t*-distribution gradually degrades to the Gaussian distribution.

To fully utilize the information from all sensors, a set $\lambda_{k}=\left\{\lambda_{1,k},\lambda_{2,k},\ldots ,\lambda_{M,k}\right\}$ is defined for the weight assigned to each sensor at time *k*. Since samples are collected sequentially over time, the measurement from the *m*-th sensor at time *k* is weighted, yielding a corrected value ${\bar{y}}_{k}^{m}=\lambda_{m,k}y_{k}^{m}$. This corrected measurement is assumed to be Gaussian distributed as $N(\lambda_{m,k}{\left(x_{k}^{m}\right)}^{T}\theta ,\lambda_{m,k}^{2}\sigma_{m}^{2}/r_{m,k})$. The set ${\bar{Y}}_{k}=\left\{{\bar{y}}_{k}^{1},{\bar{y}}_{k}^{2},\ldots ,{\bar{y}}_{k}^{M}\right\}$ represents the weighted output values from the *M* sensors at time *k*.

**Figure 1:**
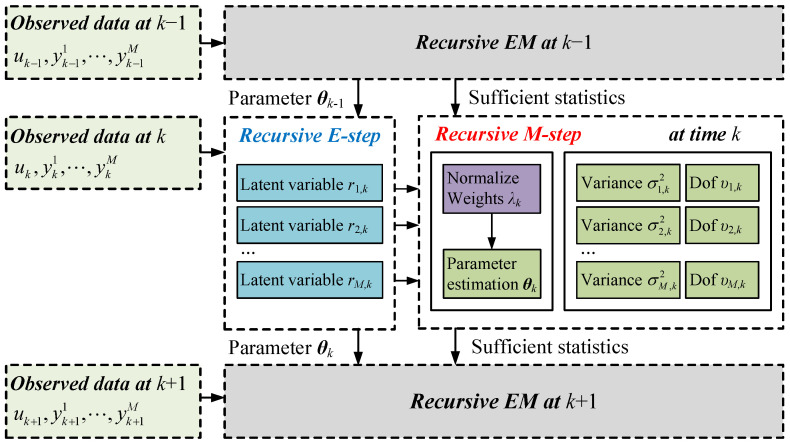
Framework of robust multi-sensor recursive EM algorithm.

Based on the above definition, when measurements data arrive sequentially, the optimization objective is formulated in the maximum likelihood sense as

(4) $${\hat{\theta }}_{k}=\arg\max_{\theta }f\left({\bar{Y}}_{k}|\theta ,\lambda_{k}\right),\;s.t.\sum_{m=1}^{M}\lambda_{m,k}=1,$$

where $f({\bar{Y}}_{k}|\theta ,\lambda_{k})$ is log-likelihood function (for details, see [23]). Solving (4), we can get $\lambda_{m,k}$ and the parameter ${\hat{\theta }}_{k}$. However, due to the unknown variances and dof introduced by Student’s *t*-distribution, $\lambda_{m,k}$ and ${\hat{\theta }}_{k}$ cannot be directly obtained, which will be addressed with a recursive EM scheme in the following sections. Figure 1 illustrates the general framework of the RMSREM algorithm.

## 3. Parameter Estimation via Robust Multi-Sensor Recursive EM Algorithm

The EM framework is widely used to estimate systems containing unobserved variables based on the maximum likelihood principle. Two core steps are composed in EM: the Expectation step (E-step) and the Maximization step (M-step), which are executed iteratively. In the E-step, the expectation of the complete-data log-likelihood function regarding the missing data needs to be calculated, and this expected value is also known as the *Q*-function. The specific calculation formula of *Q*-function is given by

(5) $$Q\left(\Theta ,\Theta^{\prime }\right)=E_{C_{\mathrm{mis}}|C_{\mathrm{obs}},\Theta^{\prime }}\left\{\log p(C_{\mathrm{obs}},C_{\mathrm{mis}}|\Theta )\right\},$$

where $C_{\mathrm{obs}}$ represents the observed variable set, $C_{\mathrm{mis}}$ denotes the unobserved (i.e., missing) variable set, Θ and $\Theta^{\prime }$ respectively stand for the parameter set to be estimated in the current iteration and obtained from the previous iteration. Then, the parameter set Θ is updated by maximizing (5) in the M-step, which can be expressed as

(6) $$\Theta =\arg\max_{\Theta }Q\left(\Theta ,\Theta^{\prime }\right).$$

Typically, the batch EM algorithm (BEM) is an iterative method. By iteratively performing the above two steps, the algorithm will gradually converge to a local maximum of the *Q*-function. For the recursive EM algorithm, the key is to convert the iterative calculation process into a recursive form. The specific implementation details of the *Q*-function recursion will be elaborated in the following sections.

### 3.1. Derivation of the Recursive Q-Function

In the batch EM, all historical data within a time period would be included in parameter estimation. When new samples arrive, the BEM requires a complete re-update, resulting in a slow response to dynamic changes in the process. This drawback limits the application of the BEM. Alternatively, a robust recursive EM is designed in this paper, where the *Q*-function is calculated in a recursive manner.

For the robust multi-sensor identification problem, the observed variable set $C_{\mathrm{obs}}$ includes input *U* and output $\bar{Y}$, i.e., $C_{\mathrm{obs}}=\left\{\bar{Y},U\right\}$, where $U=\{u_{1},\ldots ,u_{N}\}$ and $\bar{Y}=\left\{{\bar{Y}}_{1},\ldots ,{\bar{Y}}_{N}\right\}$. The unobserved variable set $C_{\mathrm{mis}}$ consists of variance scaling factors *R* induced by Student’s *t*-distribution, i.e., $C_{\mathrm{mis}}=\left\{R\right\}$, where $R=\{R_{1},\ldots ,R_{N}\}$ denotes the set of variance scaling factors for all sensors over the entire sampling period, and $R_{N}=\left\{r_{1,N},\ldots ,r_{M,N}\right\}$. Additionally, the parameter set to be estimated for this issue is $\Theta =\{\theta ,\sigma_{m}^{2},\nu_{m}\}$. The log-likelihood of the complete dataset can be decomposed using the chain rule of probability as follows:

(7) $$\begin{array}{cc} \log p(C_{\mathrm{obs}},C_{\mathrm{mis}}|\Theta ) & =\log p\left(\bar{Y}|R,U,\Theta \right)p\left(R|U,\Theta \right)p\left(U|\Theta \right)\\ \; & =\sum_{k=1}^{N}\left\{\sum_{m=1}^{M}\left[\log p\left({\bar{y}}_{k}^{m}|x_{k}^{m},r_{m,k},\Theta \right)+\log p\left(r_{m,k}|\nu_{m}\right)\right]\right\}+\log K, \end{array}$$

where ${\bar{y}}_{k}^{m}$ denotes one realization of the measurement random variable $\bar{Y}$. Given the ARX model structure with parameter set Θ, the output ${\bar{y}}_{k}^{m}$ is determined jointly by the regressive vector $x_{k}^{m}$ and the variance scaling factor $r_{m,k}$. In contrast, the factor $r_{m,k}$ is only governed by the degrees of freedom $\nu_{m}$. Since the system input *U* is an artificially generated excitation signal that does not depend on Θ, the term K=p(U|Θ) is treated as a constant. Under these considerations, the *Q*-function associated with the BEM is expressed as

(8) $$\begin{array}{cc} Q(\Theta |\Theta^{\prime }) & =E_{R|\bar{Y},U,\Theta^{\prime }}\left\{\sum_{k=1}^{N-1}\left\{\sum_{m=1}^{M}\left[\log p\left({\bar{y}}_{k}^{m}|x_{k}^{m},r_{m,k},\Theta \right)+\log p\left(r_{m,k}|\nu_{m}\right)\right]\right\}\right\}\\ \; & \;+E_{R|\bar{Y},U,\Theta^{\prime }}\left\{\sum_{m=1}^{M}\left[\log p\left({\bar{y}}_{N}^{m}|x_{N}^{m},r_{m,N},\Theta \right)+\log p\left(r_{m,N}|\nu_{m}\right)\right]\right\}. \end{array}$$

Next, (8) is rewritten into the following summation form:

(9) $$Q\left(\Theta |\Theta^{\prime }\right)=\sum_{k=1}^{N-1}q_{k}\left(\Theta |\Theta^{\prime }\right)+q_{N}\left(\Theta |\Theta^{\prime }\right).$$

In BEM, $q_{k}\left(\Theta |\Theta^{\prime }\right)$ is the expected value of the complete data logarithmic likelihood of the *k*-th sample, with $\Theta^{\prime }$ representing the current parameter estimation. Mathematically, this expectation is detailed as

(10) $$\begin{array}{cc} q_{k}\left(\Theta |\Theta^{\prime }\right) & =\sum_{m=1}^{M}\int_{r_{m,k}}p\left(r_{m,k}|C_{\mathrm{obs}},\Theta^{\prime }\right)\log p\left({\bar{y}}_{k}^{m}|x_{k}^{m},r_{m,k},\Theta \right)dr_{m,k}\\ \; & \;+\sum_{m=1}^{M}\int_{r_{m,k}}p\left(r_{m,k}|C_{\mathrm{obs}},\Theta^{\prime }\right)\log p\left(r_{m,k}|\nu_{m}\right)dr_{m,k}. \end{array}$$

Correspondingly, $q_{N}\left(\Theta |\Theta^{\prime }\right)$ is the expectation for the *N*-th data instance, which has the same expression as above.

In the context of online identification, the *Q*-function is not computed in batch for the entire dataset. Instead, it is incrementally updated by incorporating the most recent data points. This leads to a quasi-recursive formulation of the *Q*-function, expressed as

(11) $$Q_{k}\left(\Theta |\Theta_{k-1}\right)=\sum_{i=1}^{k-1}{\tilde{q}}_{i}\left(\Theta |\Theta_{i-1}\right)+{\tilde{q}}_{k}\left(\Theta |\Theta_{k-1}\right),$$

where Θ is the parameter set generated from successive recursive updates. The quantity ${\tilde{q}}_{i}\left(\Theta |\Theta_{i-1}\right)$ is defined as the posterior expectation of the complete log-likelihood for the *i*-th data point, conditioned on the parameter set $\Theta_{i-1}$ estimated at time i−1. Correspondingly, ${\tilde{q}}_{k}\left(\Theta |\Theta_{k-1}\right)$ has the same definition at time *k*. This quantity can be written explicitly as

(12) $$\begin{array}{cc} {\tilde{q}}_{i}\left(\Theta |\Theta_{i-1}\right) & =\sum_{m=1}^{M}\int_{r_{m,i}}p\left(r_{m,i}|C_{\mathrm{obs}},\Theta_{i-1}\right)\log p\left({\bar{y}}_{i}^{m}|x_{i}^{m},r_{m,i},\Theta \right)dr_{m,i}\\ \; & \;+\sum_{m=1}^{M}\int_{r_{m,i}}p\left(r_{m,i}|C_{\mathrm{obs}},\Theta_{i-1}\right)\log p\left(r_{m,i}|\nu_{m}\right)dr_{m,i}. \end{array}$$

The recursive *Q*-function at time step *k* can be formulated as follows, on the foundation of the quasi-recursive *Q*-function:

(13) $${\tilde{Q}}_{k}\left(\Theta |\Theta_{k-1}\right)=\frac{1}{k}Q_{k}\left(\Theta |\Theta_{k-1}\right).$$

Substituting (11) into (13), the recursive *Q*-function can then be transformed into:

(14) $${\tilde{Q}}_{k}\left(\Theta |\Theta_{k-1}\right)=\left(1-\frac{1}{k}\right){\tilde{Q}}_{k-1}\left(\Theta |\Theta_{k-2}\right)+\frac{1}{k}{\tilde{q}}_{k}\left(\Theta |\Theta_{k-1}\right).$$

In this paper, the standard step size 1/k can be replaced by a synthetic step size $\gamma_{k}\in R^{1}$. As demonstrated in [10], convergence is guaranteed under the conditions that the step sizes satisfy $\sum_{k=1}^{\infty }\gamma_{k}=\infty$ and $\sum_{k=1}^{\infty }\gamma_{k}^{2}<\infty$. Consequently, the final recursive form of the *Q*-function is given by

(15) $${\tilde{Q}}_{k}\left(\Theta |\Theta_{k-1}\right)=\left(1-\gamma_{k}\right){\tilde{Q}}_{k-1}\left(\Theta |\Theta_{k-2}\right)+\gamma_{k}{\tilde{q}}_{k}\left(\Theta |\Theta_{k-1}\right).$$

At the initial point of the algorithm, where k=1, the recursive *Q*-function is ${\tilde{Q}}_{1}\left(\Theta |\Theta_{0}\right)={\tilde{q}}_{1}\left(\Theta |\Theta_{0}\right)$, where $\Theta_{0}$ is an artificially assigned initial parameter set. When recursively derived from k=1 to the current step *k*, the evolution of ${\tilde{Q}}_{k}$ is governed by the following equation:

(16) $${\tilde{Q}}_{k}\left(\Theta |\Theta_{k-1}\right)=\left[\prod_{t=2}^{k}\left(1-\gamma_{t}\right)\right]E_{1}\left(\Theta \right)+\gamma_{k}E_{k}\left(\Theta \right)+\sum_{i=2}^{k-1}\left[\prod_{t=i+1}^{k}\left(1-\gamma_{t}\right)\right]\gamma_{i}E_{i}\left(\Theta \right),$$

where

$$\begin{array}{cc} \; & E_{1}\left(\Theta \right)={\tilde{q}}_{1}\left(\Theta |\Theta_{0}\right),\\ \; & E_{i}\left(\Theta \right)={\tilde{q}}_{i}\left(\Theta |\Theta_{i-1}\right),\\ \; & E_{k}\left(\Theta \right)={\tilde{q}}_{k}\left(\Theta |\Theta_{k-1}\right). \end{array}$$

Now, the recursive *Q*-function of the RMSREM algorithm has been formulated.

### 3.2. Posterior Expectation of Latent Variables

Next, the posterior expectation of the latent variables in (16) is considered. As previously described, the measurements, when conditioned on the variance-scaling factors, are Gaussian distributed. After extension, the logarithmic pdf for the measurement at time *k* can be written as

(17) $$\begin{array}{cc} \log p\left({\bar{y}}_{k}^{m}|x_{k}^{m},r_{m,k},\Theta \right)= & -\frac{1}{2}\log2\pi \lambda_{m,k}^{2}-\frac{1}{2}\log\sigma_{m,k}^{2}+\frac{1}{2}\log r_{m,k}\\ \; & -\frac{r_{m,k}}{2\lambda_{m,k}^{2}\sigma_{m,k}^{2}}{\left({\bar{y}}_{k}^{m}-\lambda_{m,k}{\left(x_{k}^{m}\right)}^{T}\theta_{k}\right)}^{2}. \end{array}$$

The variance weighting factor $r_{m,k}$ is assumed to follow a Gamma distribution. The logarithm of its likelihood function can be expressed as

(18) $$\log p\left(r_{m,k}|\nu_{m,k}\right)=-\log\Gamma \left(\frac{\nu_{m,k}}{2}\right)+\frac{\nu_{m,k}}{2}\log\left(\frac{\nu_{m,k}}{2}\right)+\frac{\nu_{m,k}}{2}\left(\log r_{m,k}-r_{m,k}\right)-\log r_{m,k}.$$

Exploiting the properties of conjugate priors, the posterior distribution of the variance weighting factor $r_{m,k}$ is also a Gamma distribution. Its explicit form can be written as

(19) $$r_{m,k}|\bar{Y},U,\Theta_{k-1}∼G\left(\frac{\nu_{m,k-1}+1}{2},\frac{\nu_{m,k-1}+\delta \left({\bar{y}}_{k}^{m}|\lambda_{m,k-1}{\left(x_{k}^{m}\right)}^{T}\theta_{k-1},\lambda_{m,k-1}^{2}\sigma_{m,k-1}^{2}\right)}{2}\right).$$

The derivation of posterior distribution of $r_{m,k}$ is provided in Appendix A. The Gamma distributed posterior expectation of the variance scaling factor $r_{m,k}$ can be computed and is denoted by $r_{m,k|y}^{\mathrm{old}}\in R^{1}$ as

(20) $$E_{r_{m,k}|\bar{Y},U,\Theta_{k-1}}\left\{r_{m,k}\right\}≜r_{m,k|y}^{\mathrm{old}}=\frac{\nu_{m,k-1}+1}{\nu_{m,k-1}+\delta \left({\bar{y}}_{k}^{m}|\lambda_{m,k-1}{\left(x_{k}^{m}\right)}^{T}\theta_{k-1},\lambda_{m,k-1}^{2}\sigma_{m,k-1}^{2}\right)},$$

where the subscript m,k|y indicates that $r_{m,k|y}^{\mathrm{old}}$ is computed conditioned on the measurement $y_{k}^{m}$ of time *k* and the parameters estimated at time k−1. The posterior of the log-variance-scaling factor, namely $\log r_{m,k}$, can be expressed as

(21) $$\begin{array}{cc} E_{r_{m,k}|\bar{Y},U,\Theta_{k-1}}\left\{\log r_{m,k}\right\} & =-\log\left(\frac{\nu_{m,k-1}+\delta \left({\bar{y}}_{k}^{m}|\lambda_{m,k-1}{\left(x_{k}^{m}\right)}^{T}\theta_{k-1},\lambda_{m,k-1}^{2}\sigma_{m,k-1}^{2}\right)}{2}\right)\\ \; & \;+\psi \left(\frac{\nu_{m,k-1}+1}{2}\right)\\ \; & =\log r_{m,k|y}^{\mathrm{old}}+\left[\psi \left(\frac{\nu_{m,k-1}+1}{2}\right)-\log\left(\frac{\nu_{m,k-1}+1}{2}\right)\right], \end{array}$$

where $\nu_{m,k-1}$ represents the dof previously estimated for the *m*-th sensor at time k−1, respectively. The function ψ(·) represents the digamma function, defined as $\psi \left(\cdot \right)=\Gamma^{\prime }\left(\cdot \right)/\Gamma \left(\cdot \right)$.

For brevity, more detailed information of the posterior expectation in the robust multi-sensor estimation is omitted. Up to this point, the derivation of the expectation step for RMSREM has been completed. The subsequent maximization step of RMSREM will be discussed in the next subsection.

### 3.3. Derivation of the Recursive Maximization Step

To recursively update the parameter θ, it is necessary to conduct the derivative of the recursive *Q*-function with respect to (w.r.t.) θ and set the resulting term to zero as

(22) $$\frac{\partial }{\partial \theta }{\tilde{Q}}_{k}\left(\Theta |\Theta_{k-1}\right)=0.$$

The online solution for θ is given as

(23) $${\hat{\theta }}_{k}={\left[{\left(\theta_{k}\right)}^{\mathrm{den}}\right]}^{-1}\left[{\left(\theta_{k}\right)}^{\mathrm{num}}\right],$$

where the two terms that determine the update of parameter vector, namely the sufficient statistics, are both computed via recursive processes. A recursive formula for the denominator is

(24) $${\left(\theta_{k}\right)}^{\mathrm{den}}=\left(1-\gamma_{k}\right){\left(\theta_{k-1}\right)}^{\mathrm{den}}+\gamma_{k}\sum_{m=1}^{M}\lambda_{m,k}^{2}\frac{r_{m,k|y}^{\mathrm{old}}}{\sigma_{m,k-1}^{2}}x_{k}^{m}{\left(x_{k}^{m}\right)}^{T},$$

while the numerator of the parameters is

(25) $${\left(\theta_{k}\right)}^{\mathrm{num}}=\left(1-\gamma_{k}\right){\left(\theta_{k-1}\right)}^{\mathrm{num}}+\gamma_{k}\sum_{m=1}^{M}\lambda_{m,k}^{2}\frac{r_{m,k|y}^{\mathrm{old}}}{\sigma_{m,k-1}^{2}}x_{k}^{m}y_{k}^{m}.$$

To estimate the measurement noise variance, the recursive *Q*-function is differentiated w.r.t. the standard deviation $\sigma_{m,k}$, and then the resulting term is set to zero, leading to

(26) $$\frac{\partial }{\partial \sigma_{m,k}}{\tilde{Q}}_{k}\left(\Theta ,\Theta_{k-1}\right)=0.$$

Employing a similar approach, the recursive fractional update equation for the variance $\sigma_{m,k}^{2}$ is obtained as

(27) $${\hat{\sigma }}_{m,k}^{2}={\left[{\left(\sigma_{m,k}^{2}\right)}^{\mathrm{den}}\right]}^{-1}\left[{\left(\sigma_{m,k}^{2}\right)}^{\mathrm{num}}\right],$$

and the denominator is

(28) $${\left(\sigma_{m,k}^{2}\right)}^{\mathrm{den}}=1,$$

while the numerator is updated as

(29) $${\left(\sigma_{m,k}^{2}\right)}^{\mathrm{num}}=\left(1-\gamma_{k}\right){\left(\sigma_{m,k-1}^{2}\right)}^{\mathrm{num}}+\gamma_{k}r_{m,k|y}^{\mathrm{old}}{\left(y_{k}^{m}-{\left(x_{k}^{m}\right)}^{T}\theta_{k}\right)}^{2}.$$

Similar to the updates of the parameter vector and variance, the update for the dof $\nu_{m}$ would be derived from the recursive *Q*-function in (16). The partial derivative of ${\tilde{Q}}_{k}\left(\Theta ,\Theta_{k-1}\right)$ w.r.t. $\nu_{m}$ is computed and equated to zero. This expression is in the following equation:

(30) $$\frac{\partial }{\partial \nu_{m}}{\tilde{Q}}_{k}\left(\Theta ,\Theta_{k-1}\right)=0.$$

Then, a function of $\nu_{m}$ is obtained as

(31) $$-\psi \left(\frac{\nu_{m}}{2}\right)+\log\left(\frac{\nu_{m}}{2}\right)+1+{\tilde{s}}_{m,k}=0,$$

with

(32) $${\tilde{s}}_{m,k}=\left(1-\gamma_{k}\right){\tilde{s}}_{m,k-1}+\gamma_{k}\left\{\left[\psi \left(\frac{\nu_{m,k-1}+1}{2}\right)-\log\left(\frac{\nu_{m,k-1}+1}{2}\right)\right]+\left(\log r_{m,k|y}^{\mathrm{old}}-r_{m,k|y}^{\mathrm{old}}\right)\right\}.$$

where ${\tilde{s}}_{m,k}$ is an artificially defined recursive auxiliary statistic, which facilitates the update of the degrees of freedom. In the BEM framework, $\nu_{m}$ is obtained directly by solving the associated equation of complete data. Regarding the online update step, the auxiliary statistic ${\tilde{s}}_{m,k}$ admits a recursive representation.

Solving (31) yields the estimate of the degrees of freedom of current time *k* for *m*-th sensor, i.e., ${\hat{\nu }}_{m,k}\in \left\{\nu |-\psi \left(\frac{\nu }{2}\right)+\log\left(\frac{\nu }{2}\right)+1+{\tilde{s}}_{m,k}=0\right\}$, where the subscript *k* is added explicitly for online estimation. In simulation studies, this can be computed using Matlab’s fsolve function, whereas in practical applications, standard nonlinear optimization methods may be employed.

### 3.4. Solution for Weights $\lambda_{m,k}$

As mentioned previously, the weights of sensors depend on the variance of measurement noise. After performing a recursive estimation of the noise variance based on hyperparameters, the multi-task likelihood function relating to $\lambda_{m,k}$ can be expressed in a following form:

(33) $$f\left({\bar{Y}}_{k}|\theta ,\lambda \right)\propto -\sum_{m=1}^{M}\left(\frac{1}{2}\log\frac{\lambda_{m,k}^{2}\sigma_{m,k-1}^{2}}{r_{m,k}^{\mathrm{old}}}\right)-\sum_{m=1}^{M}\frac{r_{m,k}^{\mathrm{old}}{\left({\bar{y}}_{k}^{m}-\lambda_{m,k}{\left(x_{k}^{m}\right)}^{T}\theta_{k-1}\right)}^{2}}{2\lambda_{m,k}^{2}\sigma_{m,k-1}^{2}}.$$

An intermediate solution of $\left(\lambda_{m,k}^{2}\sigma_{m,k-1}^{2}\right)$ w.r.t. (33) would be obtained via partial derivatives approach. Substituting the results back into (33), the term ${\left({\bar{y}}_{k}^{m}-\lambda_{m,k}{\left(x_{k}^{m}\right)}^{T}\theta_{k-1}\right)}^{2}$ would be eliminated in the subsequent derivation. Solving the sensor weights is then simplified as the following constrained optimization issue:

(34) $$\lambda_{m,k}=\arg\min_{\lambda_{m,k}}\sum_{m=1}^{M}\left(\frac{\lambda_{m,k}^{2}\sigma_{m,k-1}^{2}}{r_{m,k}}\right),\;s.t.\sum_{m=1}^{M}\lambda_{m,k}=1.$$

Introducing the Lagrange multiplier ζ, a Lagrange function is formulated as follows:

(35) $$g\left(\lambda_{m,k},\xi \right)=\sum_{m=1}^{M}\left(\frac{\lambda_{m,k}^{2}\sigma_{m,k-1}^{2}}{r_{m,k}}\right)+\zeta \left(\sum_{m=1}^{M}\lambda_{m,k}-1\right),$$

then, the weight of the sensor $\lambda_{m,k}$ is finally determined as

(36) $$\lambda_{m,k}=\frac{r_{m,k}}{\sigma_{m,k-1}^{2}}\left(\sum_{m=1}^{M}\frac{\sigma_{m,k-1}^{2}}{r_{m,k}}\right).$$

The solution of the sensor weight vector can be expressed into a matrix form as

(37) $$\lambda_{k}=A_{M,k}^{-1}1_{M}{\left(1_{M}^{T}A_{M,k}^{-1}1_{M}\right)}^{-1},$$

(38) $$A_{M,k}=\left[\begin{array}{cccc} \frac{\sigma_{1,k-1}^{2}}{r_{1,k}} & 0 & \ldots & 0\\ 0 & \frac{\sigma_{2,k-1}^{2}}{r_{2,k}} & \ldots & 0\\ \vdots & \vdots & \ddots & \vdots \\ 0 & 0 & 0 & \frac{\sigma_{M,k-1}^{2}}{r_{M,k}} \end{array}\right],$$

where $1_{M}={\left[1,1,\ldots ,1\right]}^{T}\in R^{M\times 1}$ is an *M*-dimensional vector.

Hence, the derivation of the robust multi-sensor recursive EM (RMSREM) algorithm for handling multi-sensor linear ARX models has been completed. The operating steps are listed in Algorithm 1.

**Algorithm 1.** Robust multi-sensor recursive EM algorithm.
**Require:** 
Observations of sensors $y_{1:k}^{m}$, regressive vector $x_{1:k}^{m}$;**Ensure:** 
Updated ${\hat{\theta }}_{k}$, $\sigma_{m,k}^{2}$, $\nu_{m,k}$ for a next *k*1:**Initialization**:2:    Assign step-size $\lambda_{1:k}=0.01$;3:    Assign random values in (0,1) to elements of parameter vector $\theta_{0}$;4:    Assign large values to $\sigma_{m,0}^{2}=1$;5:    Assign large values to $\nu_{m,0}=100$;6:**while** k−1→k **do**7:    **Recursive E-step (per sensor)**         Calculate the posterior expectations $E_{r_{m,k}|\bar{Y},U,\Theta_{k-1}}\left\{r_{m,k}\right\}$ via (20);         Calculate the posterior expectations $E_{r_{m,k}|\bar{Y},U,\Theta_{k-1}}\left\{\log r_{m,k}\right\}$ via (21);8:    **Weight update**         Update the sensor weights $\lambda_{m,k}$ via (37);9:    **Recursive M-step1**         Update the model parameter $\theta_{k}$ via (23);10:    **Recursive M-step2 (per sensor)**         Update the sensor noise variance $\sigma_{m,k}^{2}$ via (27);         Update the dof of Student’s *t*-distribution $\nu_{m,k}$ via (31);11:
**end while**



### 3.5. Analysis of Convergence and Computational Complexity

A. Analysis of convergence issue of RMSREM algorithm.

The Student’s *t*-distribution belongs to the curved exponential family. When only one sensor is considered, the complete-data likelihood can be decomposed as

(39) $$f\left(Y,\Theta \right)=h\left(Y,R\right)\exp\left\{-\psi \left(\theta \right)+\langle s\left(Y,R\right),\phi \left(\theta \right)\rangle \right\},$$

where the variable $s\left(Y,R\right)$ is the sufficient statistics, $\phi \left(\theta \right)$ is the natural parameters, and $\langle \cdot ,\cdot \rangle$ represents the inner product. Considering the kth time instant, the sufficient statistics can be defined as $s_{k}\left(Y,R\right)={\left[\log r_{k}-r_{k},r_{k}y_{k}^{2},r_{k}x_{k}y_{k},x_{k}x_{k}^{T}\right]}^{T}$, while the natural parameter can be defined as $\phi_{k}\left(\theta \right)={\left[\nu_{k}/2,-1/\left(2\sigma_{k}^{2}\right),-\theta_{k}/\sigma_{k}^{2},-\theta_{k}^{2}/2\sigma_{k}^{2}\right]}^{T}$. When the Kullback-Leibler divergence $K\left(g_{\theta^{*}}\left|\right|g_{\theta }\right)\overset{\Delta }{=}E_{\pi }\left[\log\left\{\frac{g\left(Y,\theta^{*}\right)}{g\left(Y,\theta \right)}\right\}\right]$ is selected as the Lyapunov function, the convergence of the online algorithm can be proved using Lyapunov stability theorem or Theorem 1 of [10], where $g\left(Y,\theta^{*}\right)$ represents the actual probability density function of the observation *Y*, and $g\left(Y,\theta \right)$ is the observed likelihood function based on estimated θ. The input variable *U* is omitted here for clarity.

The multi-sensor case would share the same convergence property with the one-sensor case, with the weights of sensors $\lambda_{m,k}$. The mathematical proof would be investigated in future work.

B. Analysis of computational complexity of RMSREM algorithm.

Considering the online updating nature of the RMSREM algorithm, the computational complexity of the one-step operation is discussed.

In the proposed algorithm, the step with the heaviest computational burden is the inversion of the denominator of parameter vector ${\left(\theta_{k}\right)}^{\mathrm{den}}\in R^{n\times n}$, which has a computational complexity of $O(n^{3})$. The computational complexity of the recursive updating of ${\left(\theta_{k}\right)}^{\mathrm{den}}\in R^{n\times n}$ is $O(m\cdot n^{2})$, involving the multiple sensors. As the sensor weight matrix is diagonal, the computational complexity of its inversion is O(m), which will be overshadowed by other steps with higher computational complexity. Moreover, the function $f\left(\nu_{m}\right)=-\psi \left(\nu_{m}/2\right)+\log\left(\nu_{m}/2\right)+1$ in (31) is monotonous regarding $\nu_{m}$. Therefore, the computational complexity of its optimal solution is O(m·i), where *i* is the iteration quantity cannot be accurately determined before the convergence of numerical optimization approach.

Therefore, the overall computational complexity of one-step operation of RMSREM algorithm when a new sample arrives is $O(n^{3}+m\cdot \left(n^{2}+i\right))$.

## 4. Algorithm Verification

### 4.1. Numerical Simulation

This experiment is a numerical simulation. To examine the multi-sensor characteristics of the RMSREM algorithm proposed in the paper, two sensors with different variances were set up and modeled using a second-order linear ARX models. The governing equation is as follows:

(40) $$y_{k}^{m}={\left(x_{k}^{m}\right)}^{T}\theta +e_{k}^{m},\;k=1,2,\ldots ,L.$$

Among them, regressive vector is set as $x_{k}^{m}={\left[y_{k-1}^{m},y_{k-2}^{m},u_{k-1},u_{k-2}\right]}^{T}$, the input $u_{k}$ follows a uniform distribution, namely $u_{k}∼U\left(-5,5\right)$, $\theta ={\left[a_{1},a_{2},b_{1},b_{2}\right]}^{T}$ is a parameter vector. Two sets of Gaussian distributed noise were generated, where $e_{k}^{1}∼N\left(0,0.001\right)$ and $e_{k}^{2}∼N\left(0,1\right)$, which were added to the noiseless output. A proportion (in this case, 5%) of the measured values are replaced with random disturbances uniformly distributed on [−3.5,−3], serving as outliers.

During the numerical simulation process, dynamic changes and multi-sensor scenarios were designed to examine the robustness of the RMSREM algorithm. To test the online recursive updating characteristics of the algorithm, the process sample sequence is split into two stages in chronological order (i.e., Phase I and Phase II). The parameter vector of the system will shift along with the switch states. The time-varying nature of parameter vectors results in the time-varying dynamics of the system, which hinders the implementation of conventional batch algorithms. The RMSREM algorithm can effectively solve the problem of system dynamic changes.

**Figure 2:**
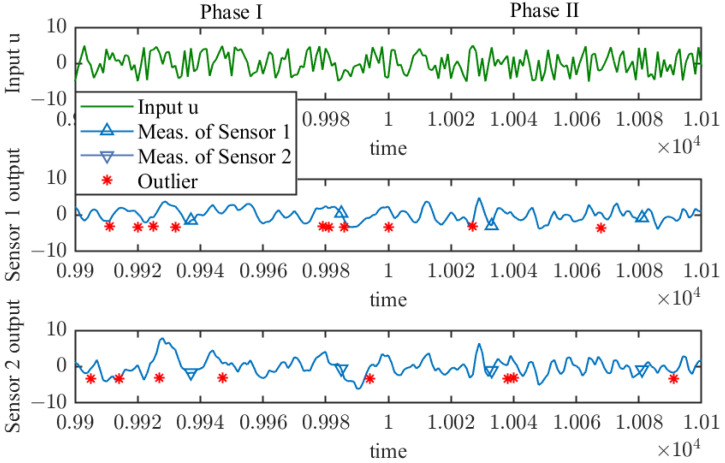
Input dual-output data (sample number: 9901 to 10,100).

As mentioned above to simulate the multi-sensor scenarios, two types of noise are generated. Subsequently, 40,000 samples are generated according to (40), add noise to the generated samplewith each sensor containing 20,000 samples and each phase containing 10,000 samples. The input dual-output curves of the numerical example near the phase transition point are shown in Figure 2, where outliers are also plotted.The time-varying actual parameter vectors are given in Table 1.

The parameter obtained from the proposed the RMSREM algorithm are also shown in Table 1, along with a comparison with the results of robust recursive EM (RREM) algorithm [15]. It should be noted that the RREM is implemented separately for the two sensors, denoted as RREM 1 and RREM 2, since it cannot process multi-sensor information. For the RMSREM algorithm, the parameter vector is obtained at the last moment of each phase. As illustrated in this table, the parameter vectors estimated by RREM 1 and the RMSREM algorithm are consistent with the actual parameter vectors. However, the parameters of RREM 2 failed to converge to the true values due to the excessively large noise variance. This indicates that the RMSREM algorithm can effectively process multi-sensor information and can estimate the actual parameter vector when some sensors are failed.

Meanwhile, to evaluate the algorithm performance, the mean square error (MSE) of the estimated output is used, which is specified as $\mathrm{MSE}=\frac{1}{N}\sum_{k=1}^{N}{\left({\hat{y}}_{k}-y_{k}\right)}^{2}$. Table 2 presents the results of self-validation (SV) and cross-validation (CV) MSE for each algorithm under different outlier ratios. In this table, CV I stands for the first stage cross-validation, while CV II stands for the second stage cross-validation. The robust batch EM (RBEM) algorithm, the recursive robust EM (RREM) algorithm, and recursive multi-task EM (RMTEM) algorithm [25] are adopted as benchmark methods. Since neither the RBEM algorithm nor the RREM algorithm can handle multi-sensor data simultaneously, they are implemented separately for the two sensors, denoted as RBEM 1, RBEM 2 and RREM 1, RREM 2, which process Sensor 1 and Sensor 2 respectively. As shown in the table, the RMSREM algorithm, RREM 1, and RBEM 1 exhibit similar performance, while RREM 2 and RBEM 2 perform poorly due to the excessively large variance of Sensor 2. In contrast, RMTEM algorithm demonstrates inferior performance under the interference of outliers.

Table 3 illustrates the time cost of the proposed the RMSREM algorithm on RTM i7-10750H CPU @ 2.60 GHz, alongside benchmark methods including the RLS algorithm, the RREM algorithm, and the RMTEM algorithm. We also designed a comparison focusing on two key indices: the time cost of one-step operation and the time cost of solving dof ν, where the latter accounts for a significant portion of the computational load. It can be seen that the solution time of the RMSREM algorithm is consistent with that of other algorithms under the recursive EM framework.

**Figure 3:**
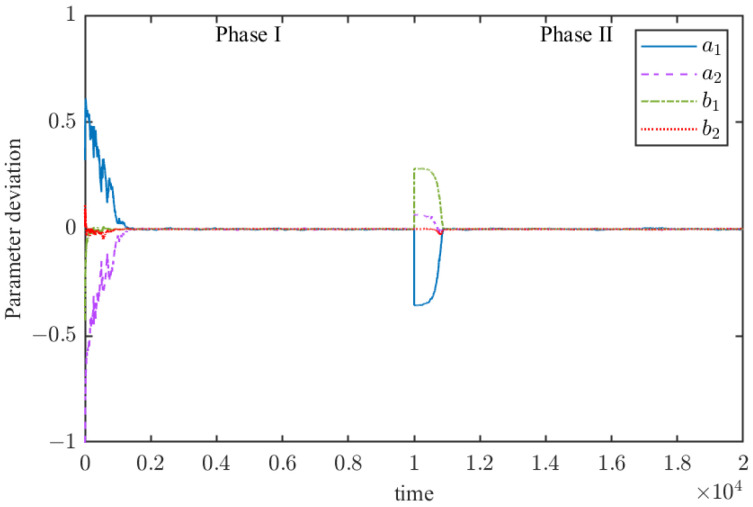
Estimate the deviation trajectory of parameters in numerical examples.

The parameter convergence process of the RMSREM algorithm is illustrated in Figure 3. The figure shows that convergence occurs after 2000 samples in the first stage. When the system switches the parameter vector to stage 2 at the 10,000-th sample, the proposed recursive algorithm converges again after 1500 samples.

**Figure 4:**
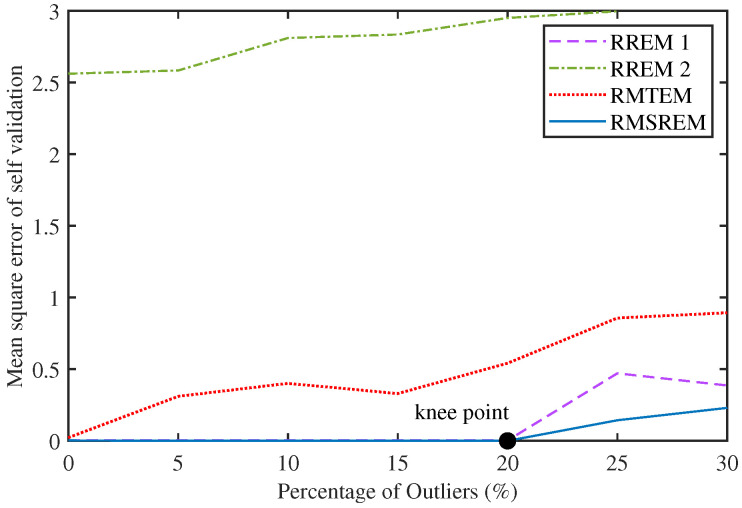
The self validation MSE of numerical examples varies with the degree of data contamination.

Furthermore, to obtain the effective range of the RMSREM algorithm against outlier interference, outliers ranging from 0% to 30% (in increments of 5%) were injected into the measured values and subjected to 50 Monte Carlo simulations. Figure 4 plots the variation curve of MSE for self-validated N-step prediction, where the knee points of the proposed algorithm are marked. The curve in the left region of the inflection point is the effective range of the algorithm in this numerical simulation. The results indicate that the inflection point of the RMSREM algorithm falls within the range of 20% to 25%.

### 4.2. Continuous Stirred Tank Reactor Example

**Figure 5:**
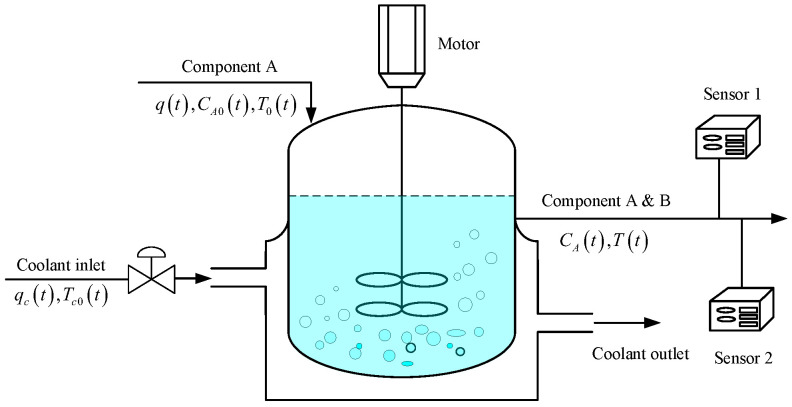
The schematic diagram of continuous stirred tank reactor.

The continuous stirred tank reactor (CSTR) is a constant-volume, exothermic, irreversible, and nonlinear system. Its dynamic behavior follows the reaction mechanism, with the core governing equations referenced from [33] as shown below (see Figure 5 for the schematic diagram):

(41) $$\frac{dC_{A}\left(t\right)}{dt}=\frac{q(t)}{V}\left(C_{A0}\left(t\right)-C_{A}\left(t\right)\right)-k_{0}C_{A}\left(t\right)\exp\left(\frac{-E}{RT(t)}\right),$$

(42) $$\begin{array}{cc} \frac{dT(t)}{dt} & =\frac{q(t)}{V}\left(T_{0}\left(t\right)-T\left(t\right)\right)-\frac{\left(-\Delta H\right)k_{0}C_{A}\left(T\right)}{\rho C_{p}}\exp\left(\frac{-E}{RT(t)}\right)\\ \; & \;+\frac{\rho_{c}C_{pc}}{\rho C_{p}V}q_{c}\left(t\right)\left\{1-\exp\left(\frac{-hA}{q_{c}\left(t\right)\rho C_{p}}\right)\right\}\left(T_{c0}\left(t\right)-T\left(t\right)\right), \end{array}$$

where $C_{A}\left(t\right)$ denotes the product concentration of Component A (the key output variable of interest in this study), and $q_{c}\left(t\right)$ represents the coolant flow rate (the key manipulated input variable). The dynamic relationship between these two variables is the focus of the analysis. Due to the nonlinear nature of the CSTR, the system needs to be linearized at preset operating points to investigate this relationship. In this work, two operating points of coolant flow rate, $q_{c}\left(t\right)=98\;L/\min$ and $q_{c}\left(t\right)=105\;L/\min$, are selected to simulate the dynamic changes of Component A concentration, thereby verifying the adaptability of the RMSREM algorithm. The definitions and nominal values of other parameters in governing equations can be found in [15].

**Figure 6:**
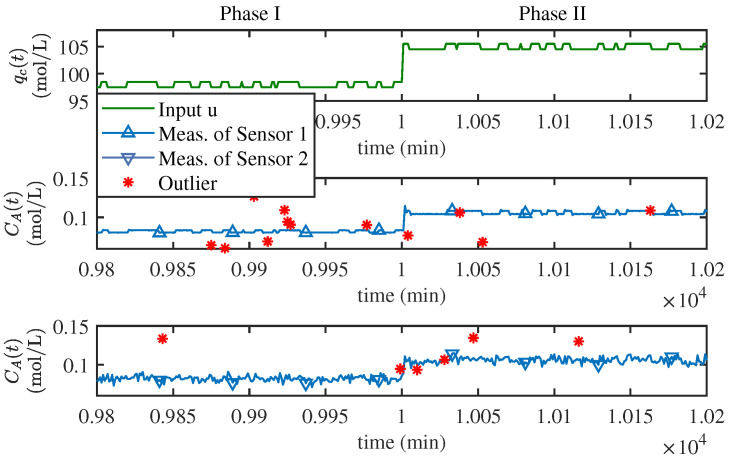
Input-dual measurements data of CSTR (sample number: 9801 to 10,200).

To guarantee sufficient excitation, a special binary signal is created that flips between −0.5 and 0.5 for the cooling liquid flow. Then, the reactor system is operated according to the governing Equations (41) and (42), and the input and output values are sampled at each minute (sampling rate: 1 min). In total, 20,000 samples are recorded, with 10,000 in each stage of the simulation. Next, two different series of Gaussian noise are added to the samples, imitating the signals coming from two separate measuring tools. The first series has a very small scatter, as $e_{k}^{1}∼N\left(0,1\times 10^{-8}\right)$. The second series has a variance about a thousand times more, i.e., $e_{k}^{2}∼N\left(0,1\times 10^{-5}\right)$. To mimic occasional bad readings, 3% of the samples are randomly picked and replaced with outliers, with a random number between −0.05 and 0.05. Figure 6 exhibits a curve segment of the input and the two sets of output neighboring the stage switching instant. The outliers that were generated on purpose are also marked in the figure.

**Figure 7:**
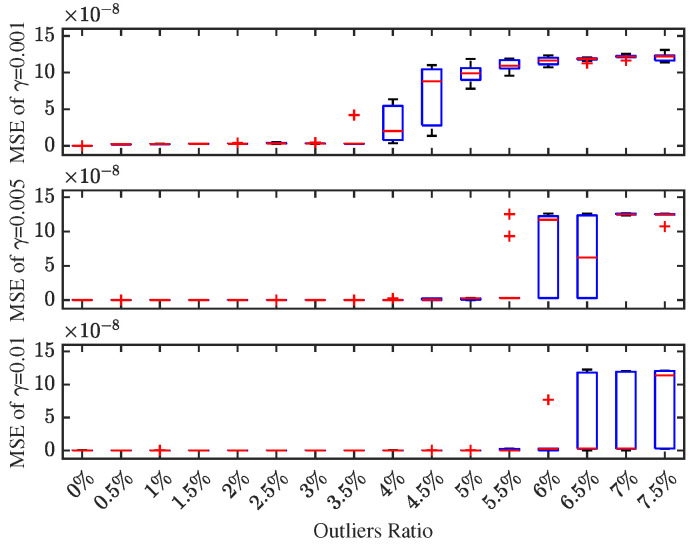
50 Monte Carlo simulations with step sizes of 0.001, 0.005, and 0.01.

The step size $\gamma_{k}$ is an artificially determined hyperparameter in the RMSREM algorithm, which significantly affects the performance of the method. To determine an appropriate step size, we conducted comparative experiments with different constant step sizes (0.001, 0.005, and 0.01) and visualized the results in Figure 7. Through comprehensive analysis of the performance metrics, the step size of 0.01 was ultimately selected for both simulation examples in this work [10,11].

**Figure 8:**
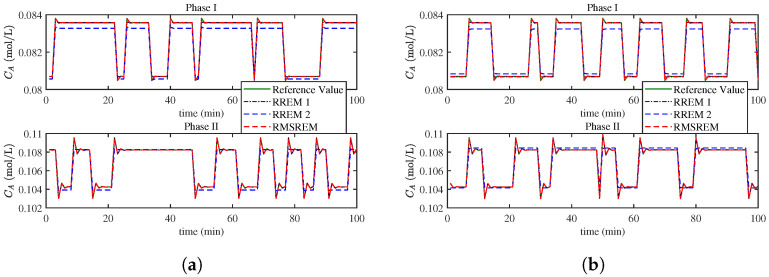
(**a**) Production concentration trajectory of component A for CSTR process in self validation. (**b**) Production concentration trajectory of component A for CSTR process in cross validation (samplin rate = 1 min).

A first-order ARX model is adopted to depict the specific dynamic characteristics. The RMSREM algorithm is then implemented and compared with the RREM algorithm. Since the RREM algorithm cannot handle multi-ensor data, it is implemented separately for each sensor, denoted as RREM 1 and RREM 2. Figure 8a shows the curve of product concentration of Component A for self-validation samples, which are the true values of samples without added noise. The corresponding curves for cross-validation are shown in Figure 8b. For the sake of clarity, only selected segments of the curves are presented, with the displayed regions chosen at random. Both SV and CV experiment involve making N-step forecasts of the production concentration of component A in CSTR. As illustrated in the figures, even in the presence of outliers, the algorithm accurately tracks the concentration trajectory in both validation scenarios. In comparison, RREM exhibits noticeable prediction errors as sensor noise levels rise.

The MSE of the algorithms, defined as $MSE=\frac{1}{N}\sum_{k=1}^{N}{\left({\hat{y}}_{k}-y_{k}\right)}^{2}$, is listed in Table 4, which shows the impact of different outlier proportions. All results are derived from 50 Monte Carlo simulations to ensure statistical reliability. The benchmark methods include the RBEM algorithm, the RREM algorithm (consistent with the numerical simulation section, implemented separately for the two sensors), and RMTEM algorithm. As illustrated in the table, the proposed the RMSREM algorithm outperforms RBEM algorithm, RREM algorithm, and RMTEM algorithm across all outlier proportion scenarios. Notably, RMTEM exhibits inferior performance under outlier interference, while RBEM algorithm and RREM algorithm also deteriorate significantly when the outlier proportion increases to 5%. In contrast, the RMSREM algorithm maintains excellent convergence capability even under such adverse conditions.

The effective range of the RMSREM algorithm in the CSTR process is explored through 50 Monte Carlo simulations. To better varify the multi-sensor robustness of the RMSREM algorithm, two methods of adding outliers are designed:

- Case 1: The outlier proportions of the two sensors are the same and increased gradually.
- Case 2: The second sensor is set to a damaged state with 100% outliers, and the outlier proportion of the first sensor is increased gradually.

**Figure 9:**
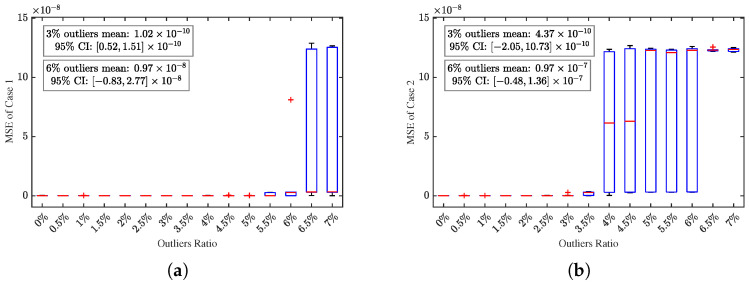
(**a**) Boxplots of MSE from 50 simulations under different outlier ratios for Case 1. (**b**) Boxplots of MSE from 50 simulations under different outlier ratios for Case 2.

The outlier proportion varies from 0% to 7.0% with an increment of 0.5%. Figure 9 presents the MSE of N-step self-validation predictions within one phase under different outlier contamination degrees. We have labeled the mean and confidence intervals of MSE for 3% and 6% outlier proportions in the graph. Specifically, Figure 9a shows that the effective range reaches 6.0% under Case 1, while Figure 9b indicates an effective range of 3.5% under the Case 2. The comparison between these two scenarios fully demonstrates the robustness of the multi-sensor framework: even when one sensor is completely compromised by outliers, the proposed the RMSREM algorithm can still leverage valid information from the other sensor to achieve accurate parameter identification.

The ability of the RMSREM algorithm method developed in this work to handle disturbances effectively is rooted in its employment of the *t*-distribution. Drawing on the properties of this distribution, the algorithm uses the posterior expectation of the variance scaling factor as a weight in the process of parameter updating. Specifically, when an unusual or erroneous measurement (an outlier) appears at time *k*, the expectation step yields a small value of $r_{m,k|y}^{\mathrm{old}}$. Consequently, the influence of the *k*-th data point is diminished during the parameter updating. Simultaneously, the contribution of each sensor is further refined with the computation of $\lambda_{m,k}$. A lower weight is assigned to the sensor that exhibits a higher degree of measurement variability (larger variance).

## 5. Discussion

Focusing on the robust identification problem of multi-sensor ARX systems in complex industrial environments, this paper proposes a robust multi-sensor recursive EM (RMSREM) algorithm. To strengthen robustness against non-Gaussian noise and outliers, the algorithm introduces the Student’s *t*-distributions to model measurement noises. An adaptive weight fusion mechanism is incorporated for multi-sensor data to mitigate the impact of single-sensor failure, and a recursive framework is adopted to achieve real-time parameter updates, adapting to the time-varying characteristics of practical systems.

Results from numerical simulations and the CSTR process case demonstrate that the proposed RMSREM algorithm achieves a lower mean square error under the same outlier contamination level compared with the RREM method. Furthermore, it maintains stable accuracy when some sensors are damaged, and can quickly track time-varying parameters when working conditions switch.

## Figures and Tables

**Table 1:** Comparison between Real Parameters of Numerical Examples and Various Algorithms (with 5% Outliers).

Indicator	Actual Value		RREM 1		RREM 2		RMSREM	
	Phase I	Phase II	Phase I	Phase II	Phase I	Phase II	Phase I	Phase II
Parm. θ	1.1430	0.7859	1.1429	0.7858	0.9336	0.6600	1.1430	0.7858
	−0.4346	−0.3679	−0.4316	−0.3673	−0.2780	−0.2936	−0.4351	−0.3665
	0.0572	0.3403	0.0572	0.3404	0.0576	0.3455	0.0578	0.3406
	0.2415	0.2417	0.2417	0.2413	0.2327	0.2573	0.2418	0.2414
Var. $\sigma_{1}^{2}$	0.001	0.001	0.0004	0.0004	0.9300	0.9635	0.0004	0.0003
Var. $\sigma_{2}^{2}$	1	1	N/A	N/A	N/A	N/A	1.0620	0.8145

N/A stands for not applicable

**Table 2:** Mean square error of the RMSREM algorithm compared to other benchmark methods.

Performance Index	SV	CV I	CV II	SV	CV I	CV II
**Outlier Percentage**	**5%**	**5%**	**5%**	**15%**	**15%**	**15%**
RBEM 1	N/A	0.0034	0.0016	N/A	0.0034	0.0018
RBEM 2	N/A	3.6222	1.7591	N/A	3.7800	1.9149
RREM 1	0.0026	0.0033	0.0017	0.0026	0.0035	0.0017
RREM 2	2.6421	3.2630	1.7452	2.7679	3.6152	1.9193
RMTEM	0.1257	0.1191	0.1322	0.5734	0.7606	0.3863
RMSREM	0.0026	0.0034	0.0017	0.0025	0.0032	0.0017

SV stands for the results of self-validation, CV I and CV II stand for the cross-validation of the first and second operating stages.

**Table 3:** The time cost of the RMSREM algorithm on RTM i7-10750H CPU @ 2.60 GHz.

	Time Cost of One-Step Operation (ms)	Time COST of Solving dof ν (ms)
RLS	0.007	N/A
RREM	0.564	0.542
RMTEM	1.227	1.144
RMSREM	1.161	1.034

**Table 4:** Comparison of MSEs of the RMSREM algorithm and benchmarks for CSTR process, (order of magnitude: $\times 10^{-7}$).

Performance Index	SV	CV I	CV II	SV	CV I	CV II
**Outlier Percentage**	**3%**	**3%**	**3%**	**5%**	**5%**	**5%**
RBEM 1	N/A	0.0581	2.2152	N/A	0.0652	2.7013
RBEM 2	N/A	0.0687	2.0692	N/A	0.0767	2.2498
RREM 1	0.0727	0.0593	0.0695	1.2713	0.0632	2.7816
RREM 2	1.3795	0.1022	0.1062	1.3943	0.1138	2.3364
RMTEM	1.7109	0.5125	2.9572	1.5573	0.6027	2.3902
RMSREM	0.0169	0.0014	0.0493	0.0170	0.0007	0.0051

SV stands for the results of self-validation, CV I and CV II stand for the cross-validation of the first and second operating stages.

## Data Availability

The data supporting the results of this research report have been detailed in the paper.

## Appendix A. Proof of (19)

According to Bayes Theorem, the posterior distribution of the variance weighting variable would be

(A1) $$p\left(R|Y,U,\Theta \right)=\frac{p(Y,U|R,\Theta )\;p(R|\Theta )}{p(Y,U|\Theta )}.$$

The denominator p(Y,U|Θ)=∫p(Y,U|R,Θ)p(R|Θ)dR, which is independent of *R* after integration and acts as a normalization constant. Considering time instant *k*, the posterior probability of $r_{m,k}$ is proportional to the following probabilities according to (A1):

(A2) $$p\left(r_{m,k}|{\bar{y}}_{k}^{m},x_{k}^{m},\Theta_{k-1}\right)\propto p\left({\bar{y}}_{k}^{m}|x_{k}^{m},r_{m,k},\Theta_{k-1}\right)\;p\left(r_{m,k}|\Theta_{k-1}\right),$$

where the first probability $p({\bar{y}}_{k}^{m}|x_{k}^{m},r_{m,k},\Theta_{k-1})$ follows a Gaussian distribution as

(A3) $$p\left({\bar{y}}_{k}^{m}|x_{k}^{m},r_{m,k},\Theta_{k-1}\right)=\frac{1}{\sqrt{2\pi }}\sqrt{\frac{r_{m,k}}{\lambda_{m,k}^{2}\sigma_{m,k-1}^{2}}}\exp\left(-\frac{r_{m,k}{\left({\bar{y}}_{k}^{m}-\lambda_{m,k}{\left(x_{k}^{m}\right)}^{T}\theta_{k-1}\right)}^{2}}{2\lambda_{m,k}^{2}\sigma_{m,k-1}^{2}}\right),$$

and the prior $p(r_{m,k}|\Theta_{k-1})$ follows a Gamma distribution as

(A4) $$p\left(r_{m,k}|\Theta_{k-1}\right)=\frac{{\left(\frac{\nu_{m,k-1}}{2}\right)}^{\frac{\nu_{m,k-1}}{2}}r_{m,k}^{\left(\frac{\nu_{m,k-1}}{2}-1\right)}}{\Gamma \left(\frac{\nu_{m,k-1}}{2}\right)}\exp\left(-\frac{\nu_{m,k-1}}{2}r_{m,k}\right).$$

Then, the posterior distribution of the variance weighting variable would be derived as

(A5) $$\begin{array}{cc} p(r_{m,k}|{\bar{y}}_{k}^{m},x_{k}^{m},\Theta_{k-1}) & \propto p\left({\bar{y}}_{k}^{m}|x_{k}^{m},r_{m,k},\Theta_{k-1}\right)\;p\left(r_{m,k}|\Theta_{k-1}\right)\\ \; & \propto {r_{m,k}}^{\left(\frac{\nu_{m,k-1}-1}{2}\right)}\exp\left(-\left(\frac{{\left({\bar{y}}_{k}^{m}-\lambda_{m,k}{\left(x_{k}^{m}\right)}^{T}\theta_{k-1}\right)}^{2}}{2\lambda_{m,k}^{2}\sigma_{m,k-1}^{2}}+\frac{\nu_{m,k-1}}{2}\right)r_{m,k}\right). \end{array}$$

Comparing with the pdf of Gamma distribution in (A4), a conclusion would be addressed that the posterior distribution of variance weighting variable is subject to a Gamma distribution as

(A6) $$r_{m,k}|\bar{Y},U,\Theta_{k-1}∼G\left(\frac{\nu_{m,k-1}+1}{2},\frac{\nu_{m,k-1}+\delta \left({\bar{y}}_{k}^{m}|{\left(x_{k}^{m}\right)}^{T}\theta_{k-1},\sigma_{m,k-1}^{2}\right)}{2}\right).$$

where $\delta \left({\bar{y}}_{k}^{m}|{\left(x_{k}^{m}\right)}^{T}\theta_{k-1},\sigma_{m,k-1}^{2}\right)≜{\left({\bar{y}}_{k}^{m}-\lambda_{m,k}{\left(x_{k}^{m}\right)}^{T}\theta_{k-1}\right)}^{2}/\left(\lambda_{m,k}^{2}\sigma_{m,k-1}^{2}\right)$ is the square of the Mahalanobis distance. Then, the posterior distribution in (19) is obtained.
